# Autonomic Symptoms in Older Adults Are Common and Associated With Health-Related Quality of Life

**DOI:** 10.3389/fneur.2021.757748

**Published:** 2021-11-23

**Authors:** Sarah Renno-Busch, Hanna Hildesheim, Janet M. T. van Uem, Ulrike Sünkel, Benjamin Röben, Kathrin Brockmann, Christian Mychajliw, Gerhard W. Eschweiler, Daniela Berg, Walter Maetzler

**Affiliations:** ^1^Center for Neurology, Department of Neurodegenerative Diseases, Hertie Institute for Clinical Brain Research, University of Tübingen, Tübingen, Germany; ^2^Department of Neurology, Christian-Albrechts University, Kiel, Germany; ^3^Department of Psychiatry and Psychotherapy, University of Tübingen, Tübingen, Germany

**Keywords:** autonomic symptoms, Composite autonomic symptom score 31 (COMPASS 31), health-related quality of life, older adults, TREND study

## Abstract

**Background:** Autonomic symptoms are common in older adults, and a large body of literature focusing on age-related diseases shows that autonomic symptoms in these diseases constrain Health-Related Quality of Life (HRQoL). To our best knowledge, the association between autonomic symptoms in older adults, independent of specific diseases, and HRQoL has not yet been assessed.

**Aim:** To assess the frequency and the effect of autonomic symptoms in general, as well as orthostatic intolerance, vasomotor, secretomotor, gastrointestinal, bladder, and pupillomotor symptoms, on HRQoL in older adults.

**Methods:** Cross-sectional data of the fourth visit of the Tübinger evaluation of Risk factors for Early detection of Neurodegeneration (TREND) study were included. Autonomic symptoms, as assessed with the Composite Autonomic Symptom Score 31 (COMPASS 31), were quantified and compared with HRQoL, as assessed with the EuroQol five-level version (EQ-5D-5L). Statistical analyses included Spearman's rank correlation and multiple linear regression analysis.

**Results:** The analysis included 928 participants with a median of 68 years; 47% were women. Of those, 85% reported at least one autonomic symptom. Gastrointestinal and secretomotor symptoms were most common. The COMPASS 31 total score and all subdomains were significantly associated with reduced HRQoL. Among the subdomains, the strongest correlations with HRQoL were found for gastrointestinal and bladder symptoms. Overall, autonomic symptoms alone explained 20% of the variance of HRQoL; when depressive mood was added, the model explained 32%.

**Conclusion:** Autonomic symptoms are associated with HRQoL and depressive symptoms in older adults.

## Introduction

The autonomic nervous system deteriorates with age at a structural and functional level and includes orthostatic intolerance, vasomotor, secretomotor, gastrointestinal, bladder, and pupillomotor symptoms. Oxidative stress, free radicals, and dysregulation of the calcium homeostasis may be main culprits at a molecular level (1). At the hormonal level, norepinephrine in the blood—a surrogate marker for the basal activity of the sympathetic nervous system—increases during adult life by about 1% per year (2), and the sensitivity of β-adrenergic receptors, regulating cardiac and vascular adaptation responses, decreases (3). Structural changes explaining autonomic symptoms have been described in detail during the last years specifically in the gut, with the activation of a senescence-associated phenotype and neurodegenerative changes being involved in these pathological processes (1). Clinical and prevalence studies have shown increasing prevalence rates with age for orthostatic intolerance (4), constipation (5, 6), lower urinary tract symptoms (7, 8), sexual dysfunction (9), and secretomotor symptoms (10, 11).

Autonomic symptoms lead to physical and emotional discomfort and limitations in activities of daily living and societal participation (12), eventually affecting Health-Related Quality of Life (HRQoL). Incontinence can lead to social isolation (13), and it is associated with depression and anxiety disorders (14). Moreover, constipation is found to be a risk factor for increased mortality in older woman (15). Numerous studies investigating patients with age-related diseases provide overwhelming evidence for the association between the presence of autonomic symptoms and reduced HRQoL (16–21). For example, patients with multiple sclerosis experiencing bladder and sexual dysfunction report a significantly lowered HRQoL, compared to patients without these symptoms (16). The presence of autonomic symptoms—assessed with the *Composite Autonomic Symptom Score 31* (COMPASS 31)—in patients with multiple sclerosis is significantly correlated with a lower physical (*r* = −0.60) and mental (*r* = −0.54) HRQoL (17). Similar results are found in patients with autonomic diabetic neuropathy due to type II diabetes mellitus; cardiac and vascular autonomic dysfunctions, including changes in blood pressure and heart rate variability, are associated with lower HRQoL and explain, together with depressive symptoms, anxiety and symptoms of peripheral neuropathy, 42–68% of the variance of HRQoL (18). A prospective controlled study, investigating patients with vascular dementia, Parkinson's disease (PD) with dementia and dementia with Lewy bodies, found positive associations with up to 45% explained variance between the presence of autonomic symptoms and poorer outcomes in different domains of HRQoL (19). In particular in PD, the association of autonomic symptoms with HRQoL has been investigated; gastrointestinal, urological, cardiovascular, visual, and thermoregulatory symptoms correlated with r values between 0.37 and 0.68 with HRQoL (20). In PD, autonomic symptoms in general, as well as specific autonomic symptoms (gastrointestinal, urinary-sexual, cardiovascular and thermoregulatory), were negatively associated with HRQoL with odds ratios between 2.5 and 3.5 (21).

Surprisingly, the effect of autonomic symptoms in older adults, not associated with a particular (age-associated) disease, is scarcely investigated. We found only studies investigating the effect of single *diseases and dysfunctions* associated with autonomic symptoms, and their influence on HRQoL. For example, it has been found that constipation affects HRQoL to a similar extent as severe chronic diseases, such as osteoarthritis, rheumatoid arthritis, allergies, and diabetes (22). HRQoL is reduced by 24% in adults over 65 years with orthostatic intolerance, compared to those without this symptom (23). Dry mouth due to xerostomia and hyposalivation explained 17% of the HRQoL variance in adults with a mean age of 85 years (24). The severity of lower urinary tract symptoms has been shown to be negatively correlated with HRQoL, in men aged between 50 and 89 (25).

However, we are not aware of any study that assessed the association of autonomic symptoms, *in general and broken down into subdomains*, with HRQoL in older adults. The COMPASS 31 is a validated questionnaire that can assess and quantify autonomic symptom severity (26). It covers six different autonomic domains. Due to this structure of the questionnaire, it is possible to assess the association of autonomic symptoms with HRQoL on a domain level, which goes beyond previous assessments investigating the associations between only single symptoms and HRQoL.

This prospective cross-sectional study assessed, in a group of older adults located in Southern Germany, the frequency of overall autonomic symptoms as well as single frequencies of orthostatic intolerance, vasomotor, secretomotor, gastrointestinal, bladder, and pupillomotor symptoms. Additionally, we investigated the association between autonomic symptoms in general as well as specific autonomic symptoms and HRQoL. Moreover, we explored the effect of depressive mood on the relationship between autonomic symptoms and HRQoL.

## Materials and Methods

### Study Design

The Tübinger evaluation of Risk factors for Early detection of Neurodegeneration (TREND study, www.trend-studie.de) is a prospective longitudinal observational cohort study currently in its sixth assessment round (performed every 2nd year) and 11th year. The major aim is to define and validate risk factors and prodromal markers for developing neurodegenerative diseases such as PD and Alzheimer's disease. This study has been including up to 1,201 older individuals with and without specific risk factors for neurodegeneration [hyposmia, depression, REM sleep behavior disorder (RBD)]. Details about the study protocol and inclusion and exclusion criteria are published (27). In brief, participants of 50 years and older, with and without history of depression, and/or hyposmia and/or RBD were included. Exclusion criteria were neurological or psychiatric disorders other than depression and RBD, cognitive deficits or dementia, current and/or history of dependency, use of neuroleptics, valproate or benzodiazepines, and severe mobility limitations. The study cohort represents an aging community in Southern Germany in an almost representative manner. This study was approved by the Ethics Committee of the University Hospital Tübingen (No 90/2009BO2), and all participants provided written informed consent.

In the fourth visit of this study (02/2015–11/2016), the COMPASS 31 (26) was included in the assessment battery for the first time, and data from this visit are presented here. From the 957 participants that took part in this visit, 29 participants were excluded from the analyses due to the following reasons: 11 had PD based on UK Brain Bank Criteria (28), seven suffered from major depression (29), three had a diagnosis of dementia according to the International Classification of Diseases, Tenth Revision (ICD-10), six did not fill out the EuroQol five-level version of EQ-5D (EQ-5D-5L), one participant did not fill out the COMPASS 31, and another one did not complete the Beck Depression Inventory (BDI-I). Details are presented in Table 1.

**Table 1:** Demographic and clinical data.

		**Median**	**Interquartile range**	**Min**.	**Max**.	**Frequency of symptoms [percent]**
Age [years]		68	10	53	87	
Gender (female) [*n*]	440					
Education period [years]		13	4	9	22	
MOCA		26	4	13	30	
COMPASS 31 (0–100)		8	15	0	75	85
Orthostatic intolerance (0–10)		0	3	0	10	27
Vasomotor (0–6)		0	0	0	6	6
Secretomotor (0–7)		0	2	0	6	50
Gastrointestinal (0–28)		1	4	0	18	54
Bladder (0–9)		0	1	0	6	43
Pupillomotor (0–15)		0	4	0	15	36
EQ VAS (0–100)		80	20	5	100	
BDI-I (0–63)		4	6	0	36	

*Data of 928 participants are presented with median, range, minimal, and maximal values and for the COMPASS 31 also presence of symptoms (defined as a score >0). BDI-I, Beck Depression Inventory; COMPASS 31, Composite Autonomic Symptom Score 31; EQ VAS, Visual analog scale of the EuroQol five-level version of EQ-5D; MoCA, Montreal Cognitive Assessment*.

### Autonomic Assessment

Autonomic function was assessed with the COMPASS 31 (26). It has a good reliability, as well as good internal and external validity as shown for healthy adults [405 individuals between 8 and 79 years (median age 32)] (26), patients with neurogenic autonomic failure and autonomic neuropathy (30), and patients with and without small fiber neuropathy (mean age 42 years, range 16–69) (31). It was previously used for patients with diabetic neuropathy (mean age 55 years) (32) and multiple sclerosis (mean age 48) (17). The participants completed the COMPASS 31 themselves, the study researchers assisted in case of need. The questionnaire consists of 31 items, covering the following six autonomic symptom domains: orthostatic intolerance (four items), vasomotor (three items), secretomotor (four items), gastrointestinal (12 items), bladder (three items), and pupillomotor (five items). Each domain consists of items asking about the occurrence, localization, frequency, severity, and time course of a symptom. Each item gets a score between 0 (“no” or “never)” and three (“constantly,” “hard,” “it gets much worse)”. The sum of each domain is multiplied by a weighting factor depending on the number of questions and the relevance of the individual areas. Adding the weighted point sums gives a value between 0 and 100. The total COMPASS 31 score gives an overview of presence and severity of individual autonomic symptoms, where 0 indicates no presence and 100 maximal presence of autonomic symptoms. Literature suggests medians and ranges for healthy volunteers = 10 ± 8 (mean age 46.6 years), peripheral neuropathy = 21 ± 13 (mean age 59.5 years), and neurogenic autonomic failure = 39 ± 18 (mean age 57.4 years) (30). Several drugs have an influence on the autonomic nerve system, including beta blockers, ACE inhibitors, calcium channel blockers, alpha-1 blockers, and antihistamines (33).

### Health-Related Quality of Life Assessment

Health-Related Quality of Life assessment was assessed using the Visual Analog Scale of the EQ-5D-5L (EQ VAS) (34, 35), which was completed at home 14 to 1 day(s) before the clinical visit. It is a one-item, general, and internationally used instrument, validated for the overall population (36) as well as for a variety of diseases, including rheumatoid arthritis, stroke, and PD (37–39). Zero represents the worst imaginable health condition and 100 the best. A recently published meta-analysis showed high construct validity and moderate test–retest reliability and responsiveness for this test (40).

### Assessment of Depression Symptoms

For the assessment of depression symptoms, we used the BDI-I. This scale is a broadly used self-reporting inventory with 21 items. Higher score values indicate more severe symptoms of depression (41).

### Statistical Analysis

Data were analyzed using SPSS Statistics software (version 23, IBM). Demographic and clinical data are presented with median, interquartile range, minimum, and maximum. Distribution of outcome parameters was tested on normality using the Shapiro–Wilk test. Due to non-normally distributed parameters, we used the Spearman's rank correlation to examine the direction, strength, and significance of correlation between the total COMPASS 31 score as well as domain-specific scores, and the EQ VAS score.

A multiple linear regression analysis with stepwise selection of the variables was performed to quantify the relative contribution *R*^2^ of independent parameters (COMPASS 31 total score, COMPASS 31 domain subscores, age, gender, BDI-I) to the variance of the dependent variable (EQ VAS). The BDI-I was included due to reported clinical (42, 43) and pathophysiological (44, 45) associations between depressive mood and autonomic symptoms. We also included diseases and drugs typically associated with autonomic symptoms, as well as physical activity in a second step in the multiple linear regression analysis, to get an estimate of the contribution of these parameters to the overall model. A stepwise approach was chosen, integrating independent parameters sequentially. Multicollinearity was tested with the variance inflation factor (VIF) and the tolerance value. VIF >10 or tolerance < 0.1 indicated multicollinearity (46, 47). *P* < 0.05 was considered significant. Due to the explorative approach, no correction for multiple testing was performed. For comparison of the participants with high and low COMPASS 31 scores, we divided the cohort at the 75th percentile and used the Mann–Whitney *U*-test for age, EQ VAS, and BDI-I and the chi-square test for gender distribution, number of diagnosis, and number of drugs typically associated with autonomic symptoms.

## Results

### Participants

Table 1 provides demographic, autonomic, and HRQoL parameters of the cohort. The median age of the cohort was 68 years, 440 participants were female, and median education year was 13. All parameters were non-normally distributed. Diagnoses and drugs that are potentially associated with autonomic symptoms are presented in Supplementary Table 1.

### Frequency of Autonomic Symptoms

A score greater than zero in the total COMPASS 31 had 85% of the participants. For the subdomains, the highest frequencies were found for the gastrointestinal (54%) and secretomotor (50%) symptoms followed by bladder symptoms (43%). Further details are presented in Table 1.

### Correlations Between Autonomic Symptoms and Health-Related Quality of Life: Univariate Analysis

**Figure 1:**
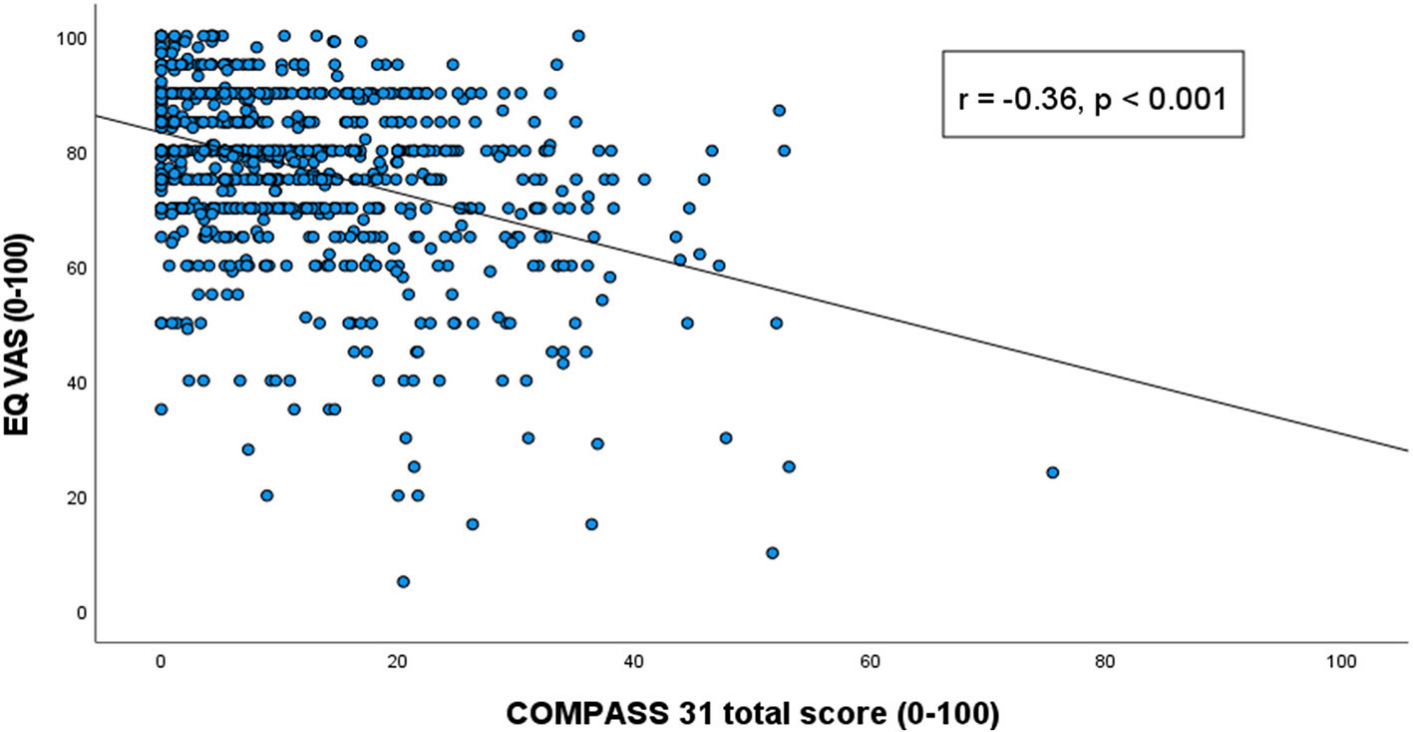
Correlation between COMPASS 31 total score and the Visual Analog Scale scores of the EuroQol five-level version of EQ-5D. Correlation values presented with Spearman's rho correlation coefficient and *p*-value for each subdomain.

**Figure 2:**
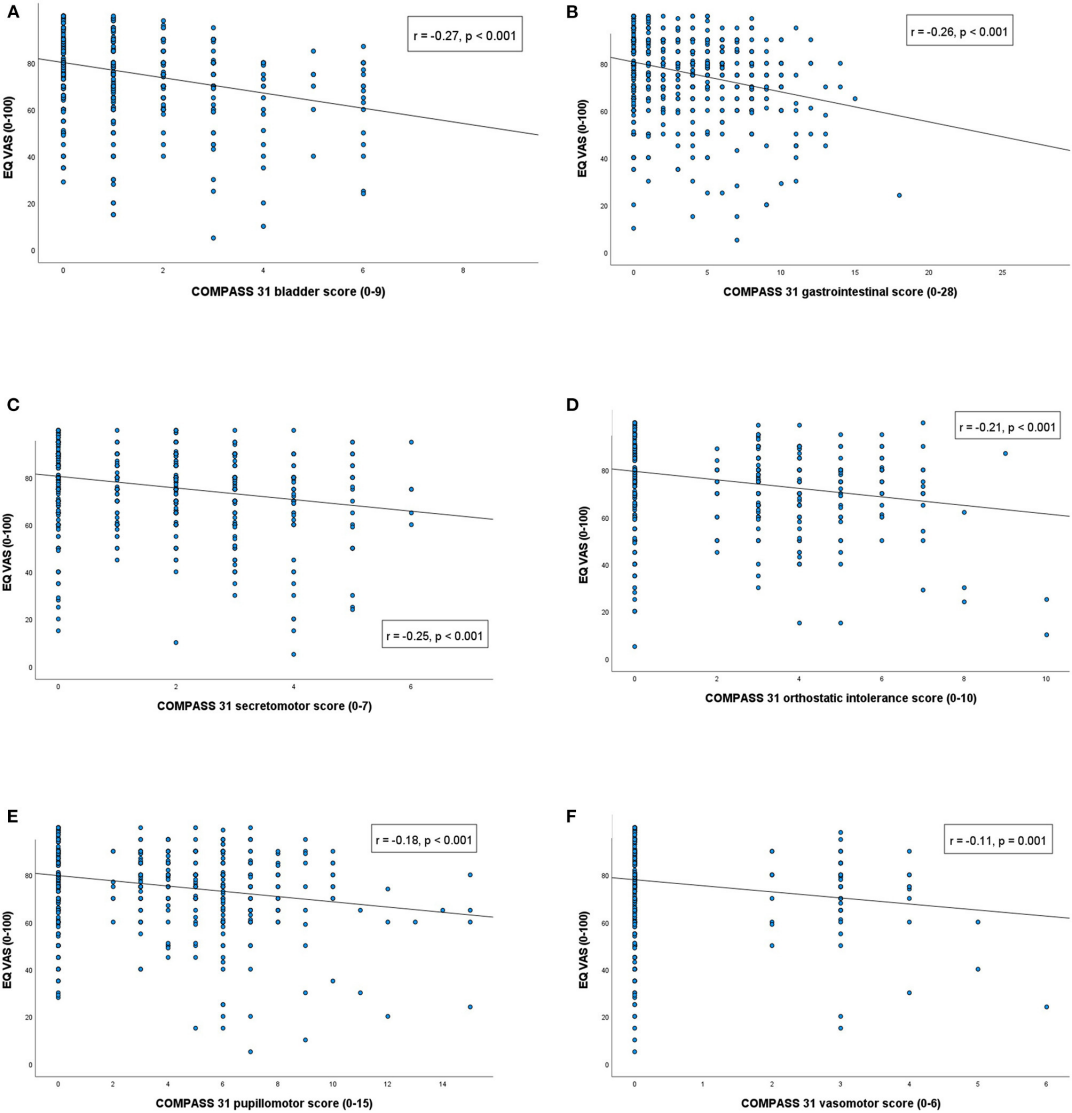
Correlation between COMPASS 31 subscores and the Visual Analog Scale scores of the EuroQol five-level version of EQ-5D. Correlation values presented with Spearman's rho correlation coefficient and *p*-value for each subdomain.

The total COMPASS 31 score correlated negatively with the EQ VAS (*r* = −0.36, *p* < 0.0005). All the COMPASS 31 subdomains also correlated significantly and negatively with the EQ VAS. The strongest correlations were found for the bladder and the gastrointestinal domains, and the lowest correlation for the vasomotor domain. Details are provided in Figures 1, 2.

### Correlations Between Autonomic Symptoms and Health-Related Quality of Life: Multivariate Analysis

To test the contribution of autonomic symptoms to HRQoL, a linear regression analysis with stepwise entry of variables with the COMPASS 31 subdomains and the EQ VAS *excluding* the BDI-I was performed. This showed a corrected *R*^2^ of 0.20 (*p* < 0.0005; 0.24 if all potential covariates were added). All six COMPASS 31 subdomains were included in the model and correlated significantly with the EQ VAS. Age was also significantly associated with the EQ VAS and was therefore included in the model. No relevant multicollinearity was found between the independent variables (VIF max = 1.17). Details are presented in Table 2.

**Table 2:** Autonomic symptoms and Health-Related Quality of Life: model without BDI-I.

	**Adjusted *R*^**2**^**	**Regression coefficient B**	**Standardized beta**	**T**	***p*-value**	**VIF**
EQ VAS	0.20					
(Constant)				22.11	**<0.0005**	
Bladder		−1.90	−0.17	−5.41	**<0.0005**	1.13
Gastrointestinal		−0.75	−0.17	−5.32	**<0.0005**	1.15
Orthostatic intolerance		−1.08	−0.15	−4.77	**<0.0005**	1.10
Secretomotor		−1.18	−0.12	−3.80	**<0.0005**	1.17
Age		−0.23	−0.11	−3.52	**<0.0005**	1.03
Pupillomotor		−0.45	−0.10	−3.00	**0.003**	1.16
Vasomotor		−1.18	−0.06	−2.11	**0.035**	1.04

*Multiple linear regression analysis corrected for the covariates age and gender (the latter was not significant and thus not included in the final model). EQ VAS, Visual analog scale of the EuroQol five-level version of EQ-5D; VIF, variance inflation factor*.

The linear regression analysis with stepwise entry of variables having the EQ VAS as dependent variable and including the COMPASS 31 subdomains and the BDI-I as independent variables produced a corrected *R*^2^ of 0.32 (*p* < 0.0005; 0.34 if all potential covariates were added). Four COMPASS 31 subdomains (bladder, gastrointestinal, orthostatic intolerance, and secretomotor) remained in the final model. The BDI-I and age also contributed to the explanation of the variance of the EQ VAS. Again, no relevant multicollinearity was observed between the independent variables (VIF max = 1.24). Details are presented in Table 3.

**Table 3:** Autonomic symptoms and Health-Related Quality of Life: model with BDI-I.

	**Adjusted *R*^**2**^**	**Regression coefficient B**	**Standardized beta**	**T**	***p*-value**	**VIF**
EQ VAS	0.32					
(Constant)				24.24	**<0.0005**	
BDI-I		−1.05	−0.40	−13.12	**<0.0005**	1.24
Bladder		−1.42	−0.13	−4.35	**<0.0005**	1.13
Gastrointestinal		−0.48	−0.11	−3.71	**<0.0005**	1.16
Age		−0.21	−0.09	−3.40	**0.001**	1.02
Orthostatic intolerance		−0.64	−0.09	−3.02	**0.003**	1.12
Secretomotor		−0.72	−0.07	−2.52	**0.012**	1.15

*Multiple linear regression analysis with the covariates age, gender, and BDI-I. BDI-I, Beck Depression Inventory; EQ VAS, Visual analog scale of the EuroQol five-level version of EQ-5D*.

### COMPASS 31 Related to the Absence/Presence of Diagnoses/Drugs Known to Impact Autonomic Function, Gender, and Age

To test the contribution of diagnoses and drugs that are already known to impact autonomic function, as well as gender and age, we compared theses parameters between participants with high COMPASS 31 scores (>75th percentile, >16.64 points) and low scores. We found that the following parameters were significantly more prevalent/higher in the latter cohort (Supplementary Table 2): EQ VAS, female gender, physical inactivity, history of cardiovascular disease, cataract, glaucoma, intake of alpha-1 blockers. No significant associations were observed for obesity, tobacco-use history, hypertension, type 2 diabetes mellitus, irritable bowel syndrome, and intake of beta and calcium channel blockers, ACE inhibitors, and antihistamines (Supplementary Table 2). To test whether the above-reported results are not only driven by study participants with severe autonomic symptoms (hence those persons who are likely to suffer from diseases associated with autonomic symptoms or who are taking medications with autonomic side effects), we performed the multiple linear regression analysis with the subgroup with the low COMPASS 31 scores only (<75th percentile, 0–16 points). We found that both models remained significant, with bladder, gastrointestinal, orthostatic intolerance, and secretomotor subscores remaining in the respective models.

## Discussion

Autonomic symptoms in a large cohort of older adults that we consider as a representative sample of an aging community in Southern Germany are very common. Only 15% of our participants experience no autonomic symptoms. Our results are in line with many studies on prevalence rates investigating particular autonomic symptoms in adults (4–11). For example, the median value of the COMPASS 31 total score (eight points) in our cohort, with a median age of 68 years, was comparable to the results published recently on a cohort with a mean age of 46.6 years (30) (10 points). Interestingly, gender did not significantly contribute to HRQoL in our analysis models. These results are in line with previous population-based studies (48–50) and emphasize the need for detailed anamnesis in clinical routine in both women and men.

Not surprisingly and in agreement with previous studies (51), also in this cohort, the COMPASS 31 scores were positively associated with the number of diagnoses and drugs that are already known for their impact on autonomic symptoms.

In these studies, a direct association between autonomic function and HRQoL has been demonstrated (16–21). An example is PD, where non-motor symptoms, especially autonomic symptoms, have a greater impact on HRQoL than motor symptoms (20, 52).

However, to our best knowledge, this is the first publication presenting an overview of the interaction between autonomic symptoms and HRQoL in a considerably large cohort of older adults not selected based on the presence of one particular disease.

Both univariate and multivariate analyses show the relevant association of autonomic symptoms with HRQoL. The two linear regression models demonstrate that the presence of autonomic symptoms (depending on the in- or exclusion of the cofactor BDI-I, see also below) explain between 20 and 32% of the variance of HRQoL. We found that bladder, gastrointestinal, and secretomotor symptoms and orthostatic intolerance correlated strongest with HRQoL. This effect continued to be significant when we did not include the 25% most severely affected according to COMPASS 31 results in the calculation, suggesting that these autonomic symptoms are also most prevalent and HRQoL-constraining in the “healthier” older population. Symptoms such as urge to urinate or orthostatic intolerance are probably perceived more disturbing compared to symptoms from other systems. The physical complaints and the possible consequences—e.g., syncope, incontinence, pain, hyperhidrosis—can be significantly restrictive and shameful for those affected, especially in a social context, and can influence one's lifestyle.

In line with other studies, urinary bladder symptoms constrained HRQoL most (25, 53, 54). High prevalence rates for lower urinary tract symptoms were found in adults over 60 years (men 81%, women 79%), based on a population-based study carried out in five countries (7). Against this background, urinary bladder symptoms are a huge burden on the healthcare system in an aging and growing population. Numerous studies also show the negative association between constipation and HRQoL (22, 55, 56). The negative effects of constipation have been shown to be most relevant for the domains general health, social functioning, and mental health, while the physical health is obviously less affected (22). Another study found higher prevalence rates for anxiety and depression (33 vs. 11%) and more problems in the discomfort and mobility dimensions, in people suffering from orthostatic intolerance, compared to those without. These effects are probably driven by dizziness, fatigue, and decreased memory (23). It has also been shown that xerostomia (24, 57) and dry eyes (58) constrain HRQoL.

Moreover, it is described that symptoms of pupillomotor (photosensitivity and difficulty to focus fast) and vasomotor (vasoconstriction on hands/feet such as Raynaud syndrome) correlate with a reduced HRQoL (59, 60). In our study, these symptoms have a lower effect on HRQoL, presumably because they are only present intermittently. Moreover, pupillomotor and vasomotor symptoms can be prevented and, at least on a symptomatic level, treated. For example, triggering factors such as cold for Raynaud syndrome or bright light for photosensitivity can be identified and exposure can be prevented by those affected.

The relationship between depression, autonomic symptoms, and HRQoL is complex. We show that HRQoL is more reduced with low mood additional to the presence of autonomic symptoms, compared to the presence of autonomic symptoms alone. The model with the cofactor current depressive mood (by BDI-I) explained 12% more variability in HRQoL, compared to the model without depressive mood. This may be, at least partly, explained by the influence that depressive mood has on the perception of autonomic symptoms (61). A recent study investigating the relationship between depressive symptoms and autonomic symptoms in multiple system atrophy showed that people with depressive symptoms scored higher on the vigilance scores, meaning that they are more focused on the perception of autonomic symptoms, even though their autonomic symptoms were not more severe compared to the non-depressive patients (61).

However, the relationship between depression, autonomic symptoms, and HRQoL may be beyond perception and more multilayered. For example, a depression itself leads to increased sympathetic activity and a decreased parasympathetic activity, affecting the heart rate (62). Moreover, depression correlates negatively with heart rate variability (42). These associations are difficult to explain simply by perception effects. The Porges' “Polyvagal Theory” emphasizes that the portion of the vagus nerve coming from the Ncl. ambiguus forms a ventral vagal complex with the nuclei of the facial nerve and trigeminal nerve, explaining the obvious interaction between mood and the autonomic system. The ventral vagal complex is responsible for vocalization, facial expression, chewing, swallowing, head rotation, and cardiac control—all essential components of attention, emotion, communication, and social behavior (44). The vagus nerve is part of the parasympathetic nervous system, of which the activity is in general reduced in depression (62). A restricted parasympathetic function leads to slowed adaptation and reaction to a new situation (63). Depressive symptoms may include lack of drive, a flattened affect, decreased empathy, hypomotor facial expressions, and changes in social behavior. These symptoms match the changes in “vagal dysfunction” (63) and may further support the theory of pathophysiologically driven associations between autonomic changes and depression. Interestingly, chronic electric stimulation of the vagus nerve in patients suffering from major depression leads to the improvement of depressive symptoms (64).

Another interesting theory with this respect, the theory of neurovisceral integration (45), suggests an interaction between mood and autonomic function *via* the respiratory sinus arrhythmia (RSA). RSA is the heart rate variability over the respiration cycle and a measure for autonomic flexibility. Rest inhibits the activation of the amygdala *via* the medial prefrontal cortex, leading to enhanced parasympathetic activation *via* the vagus nerve and, consecutively, to higher RSA levels. According to this theory, depressed people have reduced autonomic flexibility and consequently lower RSA levels (65).

In summary, these theories suggest that the autonomic nervous system and regulatory pathways of attention and emotion are intertwined, influencing each other. These complex relationships are only partially understood, and further studies are needed to improve our knowledge concerning how depressive mood affects the relationship between autonomic restrictions and HRQoL.

This study faces some limitations. First, the COMPASS 31 questionnaire asks for anamnestic data of various autonomic symptoms and reflects subjective experience and feelings. It is neither objective nor can it identify causal or disruptive factors that could potentially affect HRQoL. Second, we only used the EQ VAS and not the full EQ-5D-5L questionnaire, so that our analyses are based on a single statement about self-rated health. We decided to only use the EQ VAS because it is a semi-quantitative measure, easy and quick to use and evaluate, and validated (66). It should be noted that we did not specifically instruct subjects to complete the EQ VAS on a normal day, so it may be that some subjects completed it in an unusual life situation that we could not notice. Eventually, current depressive mood could not only directly affect HRQoL (which we tried to take into account including the BDI-I) but may have also an effect on completing the EQ VAS and could thus distort the results. In addition, we cannot make any statements about causal relationships between autonomic symptoms and HRQoL, as this is a cross-sectional evaluation.

In summary, autonomic symptoms in older adults are common. The total score of the COMPASS 31 and each of the single domains correlated significantly with HRQoL. Autonomic symptoms explained 20% (without inclusion of depressive mood) to 32% (including depressive mood) of the variation of HRQoL in our cohort. Respective models remained significant in the subgroup of 75% with the lowest COMPASS 31 scores. This is, to our best knowledge, the first study of this magnitude to investigate the association of autonomic symptoms with HRQoL in a representative sample of older adults. Our results urgently speak in favor of an in-depth and differentiated investigation and confirmation of these pilot data, with the eventual aim of effectively treating HRQoL-relevant autonomic symptoms in the older population.

## Data Availability Statement

The raw data supporting the conclusions of this article will be made available by the authors, without undue reservation.

## Ethics Statement

The studies involving human participants were reviewed and approved by Ethics Committee of the University Hospital Tübingen (No 90/2009BO2). The patients/participants provided their written informed consent to participate in this study.

## Author Contributions

SR-B, JU, and WM developed the idea. SR-B and HH performed the literature search and the statistical analysis. SR-B, JU, HH, and WM framed the outline. SR-B, JU, US, BR, KB, HH, CM, GE, DB, and WM worked on different aspects of the manuscript. All authors commented on the manuscript and approved the final version.

## Funding

This study received funding from the Hertie Institute for Clinical Brain Research, the German Center for Neurodegenerative Diseases, the Center for Integrative Neuroscience, TEVA Pharmaceutical Industries Ltd., Union Chimique Belge (UCB), Janssen Pharmaceuticals, Inc., and the International Parkinson Fonds. The funders were not involved in the study design, collection, analysis, interpretation of data, the writing of this article or the decision to submit it for publication.

## Conflict of Interest

The authors declare that the research was conducted in the absence of any commercial or financial relationships that could be construed as a potential conflict of interest.

## Publisher's Note

All claims expressed in this article are solely those of the authors and do not necessarily represent those of their affiliated organizations, or those of the publisher, the editors and the reviewers. Any product that may be evaluated in this article, or claim that may be made by its manufacturer, is not guaranteed or endorsed by the publisher.

## Abbreviations

HRQoL: Health-Related Quality of Life
TREND: Tübinger evaluation of Risk factors for Early detection of Neurodegeneration
COMPASS 31: Composite Autonomic Symptom Score 31
EQ-5D-5L: EuroQol five-level version
PD: Parkinson's disease
RBD: REM sleep behavior disorder
ICD-10, International Classification of Diseases: Tenth Revision
BDI-I: Beck Depression Inventors
VAS: Visual Analog Scale
VIF: Variance inflation factor
RSA: Respiratory sinus arrhythmia.

## References

[1] Saffrey MJ. Cellular changes in the enteric nervous system during ageing. Dev Biol. (2013) 382:344–55. 10.1016/j.ydbio.2013.03.015 23537898

[2] Ziegler MG Lake CR Kopin IJ. Plasma noradrenaline increases with age. Nature. (1976) 261:333–5. 10.1038/261333a0 1272412

[3] Davies CH Ferrara N Harding SE. Beta-adrenoceptor function changes with age of subject in myocytes from non-failing human ventricle. Cardiovasc Res. (1996) 31:152–6. 10.1016/S0008-6363(95)00187-5 8849600

[4] Ndayisaba J-P Fanciulli A Granata R Duerr S Hintringer F Goebel G. Sex and age effects on cardiovascular autonomic function in healthy adults. Clin Auton Res. (2015) 25:317–26. 10.1007/s10286-015-0310-1 26285905

[5] Mugie SM Benninga MA Di Lorenzo C. Epidemiology of constipation in children and adults: a systematic review. Best Pr Res Clin Gastroenterol. (2011) 25:3–18. 10.1016/j.bpg.2010.12.010 21382575

[6] Werth BL Williams KA Pont LG. A longitudinal study of constipation and laxative use in a community-dwelling elderly population. Arch Gerontol Geriatr. (2015) 60:418–24. 10.1016/j.archger.2015.02.004 25736738

[7] Irwin DE1 Milsom I Hunskaar S Reilly K Kopp Z Herschorn S. Population-based survey of urinary incontinence, overactive bladder, and other lower urinary tract symptoms in five countries: results of the EPIC study. Eur Urol. (2006) 50:1306–14. 10.1016/j.eururo.2006.09.019 17049716

[8] Boyle P Robertson C Mazzetta C Keech M Hobbs FDR Fourcade R. UrEpik study group. The prevalence of lower urinary tract symptoms in men and women in four centres The UrEpik study. BJU Int. (2003) 92:409–14. 10.1046/j.1464-410X.2003.04369.x 12930430

[9] Kang SY Lee JA Sunwoo S Yu B-Y Lee JH Cho CH. Prevalence of sexual dysfunction and associated risk factors in middle-aged and elderly Korean men in primary care. J Sex Res. (2016) 1–14. 10.1080/00224499.2016.1174657 27215144

[10] Bakkar MM Shihadeh WA Haddad MF Khade YS. Epidemiology of symptoms of dry eye disease (DED) in Jordan: a cross-sectional non-clinical population-based study. Cont Lens Anterior Eye. (2016) 39:197–202. 10.1016/j.clae.2016.01.003 26833214

[11] Olaniyan SI Fasina O Bekibele CO Ogundipe OA. Dry eye disease in an adult population in South-West Nigeria. Cont Lens Anterior Eye. (2016) 39:359–64. 10.1016/j.clae.2016.06.008 27396514

[12] World Health Organization. The International Classification of Functioning, Disability and Health. Geneva (2001).

[13] Yip SO Dick MA McPencow AM Martin DK Ciarleglio MM Erekson EA. The association between urinary and fecal incontinence and social isolation in older women. Am J Obstet Gynecol. (2013) 208:146.e1–146.e7. 10.1016/j.ajog.2012.11.010 23159696 PMC3715999

[14] Coyne KS Kvasz M Ireland AM Milsom I Kopp ZS Chapple CR. Urinary incontinence and its relationship to mental health and health-related quality of life in men and women in Sweden, the United Kingdom, and the United States. Eur Urol. (2012) 61:88–95. 10.1016/j.eururo.2011.07.049 21831517

[15] Koloski NA Jones M Wai R Gill RS Byles J Talley NJ. Impact of persistent constipation on health-related quality of life and mortality in older community-dwelling women. Am J Gastroenterol. (2013) 108:1152–8. 10.1038/ajg.2013.137 23670115

[16] Nortvedt MW Riise T Myhr K-M Landtblom A-M Bakke A Nyland HI. Reduced quality of life among multiple sclerosis patients with sexual disturbance and bladder dysfunction. Mult Scler J. (2001) 7:231–5. 10.1177/135245850100700404 11548982

[17] Cortez MM Nagi Reddy SK Goodman B Carter JL Wingerchuk DM. Autonomic symptom burden is associated with MS-related fatigue and quality of life. Mult Scler Relat Disord. (2015) 4:258–63. 10.1016/j.msard.2015.03.007 26008943

[18] Chyun DA Melkus GD Katten DM Price WJ Davey JA Grey N. The association of psychological factors, physical activity, neuropathy, and quality of life in type 2 diabetes. Biol Res Nurs. (2006) 7:279–88. 10.1177/1099800405285748 16581898

[19] Allan L McKeith I Ballard C Kenny RA. The prevalence of autonomic symptoms in dementia and their association with physical activity, activities of daily living and quality of life. Dement Geriatr Cogn Disord. (2006) 22:230–7. 10.1159/000094971 16902277

[20] Tomic S Rajkovaca I Pekic V Salha T Misevic S. Impact of autonomic dysfunctions on the quality of life in Parkinson's disease patients. Acta Neurol Belg. (2017) 117:207–11. 10.1007/s13760-016-0739-6 28028676

[21] Merola A Romagnolo A Rosso M Suri R Berndt Z Maule S. Autonomic dysfunction in Parkinson's disease: a prospective cohort study. Mov Disord. (2018) 33:391–7. 10.1002/mds.27268 29278286

[22] Belsey J Greenfield S Candy D Geraint M. Systematic review: impact of constipation on quality of life in adults and children. Aliment Pharmacol Ther. (2010) 31:938–49. 10.1111/j.1365-2036.2010.04273.x 20180788

[23] Kim N Park J Hong H Kong ID Kang H. Orthostatic hypotension and health-related quality of life among community-living older people in Korea. Qual Life Res. (2020) 29:303–12. 10.1007/s11136-019-02295-6 31515746

[24] Gerdin EW Einarson S Jonsson M Aronsson K Johansson I. Impact of dry mouth conditions on oral health-related quality of life in older people. Gerodontology. (2005) 22:219–26. 10.1111/j.1741-2358.2005.00087.x 16329230

[25] Arslantas D Gokler ME Unsal A Baseskioglu B. Prevalence of lower urinary tract symptoms among individuals aged 50 years and over and its effect on the quality of life in a semi-rural area of western Turkey. LUTS Low Urin Tract Symptoms. (2017) 9:5–9. 10.1111/luts.12100 28120441

[26] Sletten DM Suarez GA Low PA Mandrekar J Singer W. COMPASS 31: a refined and abbreviated composite autonomic symptom score. Mayo Clin Proc. (2012) 87:1196–201. 10.1016/j.mayocp.2012.10.013 23218087 PMC3541923

[27] Gaenslen A Wurster I Brockmann K Huber H Godau J Faust B. Prodromal features for Parkinson's disease–baseline data from the TREND study. Eur J Neurol. (2014) 21:766–72. 10.1111/ene.12382 24612314

[28] Hughes AJ Daniel SE Kilford L Lees AJ. Accuracy of clinical diagnosis of idiopathic Parkinson's disease: a clinico-pathological study of 100 cases. J Neurol Neurosurg Psychiatry. (1992) 55:181. 10.1136/jnnp.55.3.181 1564476 PMC1014720

[29] Bech P Rasmussen NA Olsen LR Noerholm V Abildgaard W. The sensitivity and specificity of the major depression inventory, using the present state examination as the index of diagnostic validity. J Affect Disord. (2001) 66:159–64. 10.1016/S0165-0327(00)00309-8 11578668

[30] Singer W Sletten D Mandrekar J Low P. Validation of a refined and abbreviated composite autonomic symptom score (S37.001). Neurology. (2013) 80:S37.001. 23218087 10.1016/j.mayocp.2012.10.013 PMC3541923

[31] Treister R O'Neil K Downs HM Oaklander AL. Validation of the composite autonomic symptom scale 31 (COMPASS-31) in patients with and without small fiber polyneuropathy. Eur J Neurol. (2015) 22:1124–30. 10.1111/ene.12717 25907824 PMC4464987

[32] Greco C Di Gennaro F D'Amato C Morganti R Corradini D Sun A. Validation of the composite autonomic symptom score 31 (COMPASS 31) for the assessment of symptoms of autonomic neuropathy in people with diabetes. Diabet Med. (2017) 34:834–8. 10.1111/dme.13310 27990686

[33] Ruška B Pavičić T Pavlović I Junaković A Adamec I Crnošija L. Performance of the COMPASS-31 questionnaire with regard to autonomic nervous system testing results and medication use: a prospective study in a real-life setting. Neurol Sci. (2018) 39:2079–84. 10.1007/s10072-018-3542-8 30140988

[34] Bergner M Bobitt RA Fanshel S Bush JW Hunt SM McEwen J. EuroQol - a new facility for the measurement of health-related quality of life. Health Policy. (1990) 16:199–208. 10.1016/0168-8510(90)90421-9 10109801

[35] Brooks R Group E Badia X Fernandez E Segura A Nord E. EuroQol: the current state of play. Health Policy. (1996) 37:53–72. 10.1016/0168-8510(96)00822-6 10158943

[36] Brazier J Jones N Kind P. Testing the validity of the Euroqol and comparing it with the SF-36 health survey questionnaire. Qual Life Res. (1993) 2:169–80. 10.1007/BF00435221 8401453

[37] Hurst NP Jobanputra P Hunter M Lambert M Lochhead A Brown H. Validity of EuroQoL—a generic health status instrument—in patients with rheumatoid arthritis. Rheumatology. (1994) 33:655–62. 10.1093/rheumatology/33.7.655 8019795

[38] Dorman PJ Waddell F Slattery J Dennis M Sandercock P. Is the EuroQol a valid measure of health-related quality of life after stroke? Stroke. (1997) 28:1876–82. 10.1161/01.STR.28.10.1876 9341688

[39] Schrag A Selai C Jahanshahi M Quinn NP. The EQ-5D–a generic quality of life measure-is a useful instrument to measure quality of life in patients with Parkinson's disease. J Neurol Neurosurg Psychiatry. (2000) 69:67–73. 10.1136/jnnp.69.1.67 10864606 PMC1737007

[40] Cheng LJ Tan RLY Luo N. Measurement properties of the eq vas around the globe: a systematic review and meta-regression analysis. Value Heal. (2021) 24:1223–33. 10.1016/j.jval.2021.02.003 34372988

[41] Beck AT Ward CH Mendelson M Moch J Erbaugh J. An inventory for measuring depression. Arch Gen Psychiatry. (1961) 4:53–63. 10.1001/archpsyc.1961.01710120031004 13688369

[42] Kemp AH Quintana DS Gray MA Felmingham KL Brown K Gatt JM. Impact of depression and antidepressant treatment on heart rate variability: a review and meta-analysis. Biol Psychiatry. (2010) 67:1067–74. 10.1016/j.biopsych.2009.12.012 20138254

[43] Dauphinot V Rouch I Kossovsky MP Pichot V Dorey J-M Krolak-Salmon P. Depressive symptoms and autonomic nervous system dysfunction in an elderly population-based study: the PROOF study. J Affect Disord. (2012) 143:153–9. 10.1016/j.jad.2012.05.045 22910448

[44] Porges SW. Orienting in a defensive world: mammalian modifications of our evolutionary heritage. Psychophysiology. (1995) 32:301–18. 10.1111/j.1469-8986.1995.tb01213.x 7652107

[45] Thayer JF Lane RD. A model of neurovisceral integration in emotion regulation and dysregulation. J Affect Disord. (2000) 61:201–16. 10.1016/S0165-0327(00)00338-4 11163422

[46] Yoo W Mayberry R Bae S Singh K Peter He Q Lillard JW. Study of effects of multicollinearity in the multivariable analysis. Int J Appl Sci Technol. (2014) 4:9–19. 25664257 PMC4318006

[47] Slinker BK Glantz SA. Multiple regression for physiological data analysis: the problem of multicollinearity. Am J Physiol. (1985) 249:R1–12. 10.1152/ajpregu.1985.249.1.R1 4014489

[48] Lima MG Barros MB de A César CLG Goldbaum M Carandina L Ciconelli RM. Health related quality of life among the elderly: a population-based study using SF-36 survey. Cad Saude Publica. (2009) 25:2159–67. 10.1590/S0102-311X2009001000007 19851616

[49] Li L Wang HM Shen Y. Chinese SF-36 health survey: translation, cultural adaptation, validation, and normalisation. J Epidemiol Community Health. (2003) 57:259–63. 10.1136/jech.57.4.259 12646540 PMC1732425

[50] Wyss K Wagner AK Whiting D Mtasiwa DM Tanner M Gandek B. Validation of the Kiswahili version of the SF-36 health survey in a representative sample of an urban population in Tanzania. Qual Life Res. (1999) 8:111–20. 10.1023/A:1026431727374 10457744

[51] Goldstein DS Cheshire WP. The autonomic medical history. Clin Auton Res. (2017) 27:223–33. 10.1007/s10286-017-0425-7 28551871 PMC8942132

[52] Martinez-Martin P Rodriguez-Blazquez C Kurtis MM Chaudhuri KR. The impact of non-motor symptoms on health-related quality of life of patients with Parkinson's disease. Mov Disord. (2011) 26:399–406. 10.1002/mds.23462 21264941

[53] Pintarelli V Perchon L Lorenzetti F Toniolo Neto J Dambros M. Elderly men's quality of life and lower urinary tract symptoms: an intricate relationship. Int Braz J Urol. (2011) 37: 10.1590/S1677-55382011000600012 22234010

[54] Girman CJ Jacobsen SJ Tsukamoto T Richard F Garraway WM Sagnier P-P. Health-related quality of life associated with lower urinary tract symptoms in four countries. Urology. (1998) 51:428–36. 10.1016/S0090-4295(97)00717-6 9510348

[55] Norton C. Constipation in older patients: effects on quality of life. Br J Nurs. (2006) 15:188–92. 10.12968/bjon.2006.15.4.20542 16603983

[56] Tvistholm N Munch L Danielsen AK. Constipation is casting a shadow over everyday life-a systematic review on older people's experience of living with constipation. J Clin Nurs. (2016) 26:902–14. 10.1111/jocn.13422 27271918

[57] Niklander S Veas L Barrera C Fuentes F Chiappini G Marshall M. Risk factors, hyposalivation and impact of xerostomia on oral health-related quality of life. Braz Oral Res. (2017) 31:e14. 10.1590/1807-3107bor-2017.vol31.0014 28099580

[58] Miljanović B Dana R Sullivan DA Schaumberg DA. Impact of dry eye syndrome on vision-related quality of life. Am J Ophthalmol. (2007) 143:409–15.e2. 10.1016/j.ajo.2006.11.060 17317388 PMC1847608

[59] De Angelis R Salaffi F Grassi W. Health-related quality of life in primary raynaud phenomenon. JCR J Clin Rheumatol. (2008) 14:206–10. 10.1097/RHU.0b013e31817a2485 18766120

[60] Hughes M Snapir A Wilkinson J Snapir D Wigley FM Herrick AL. Prediction and impact of attacks of Raynaud's phenomenon, as judged by patient perception. Rheumatology. (2015) 54:1443–7. 10.1093/rheumatology/kev002 25752312

[61] Martinez J Palma J-A Norcliffe-Kaufmann L Garakani A Kaufmann H. Impact of depressive symptoms on self-perceived severity of autonomic dysfunction in multiple system atrophy: relevance for patient-reported outcomes in clinical trials. Clin Auton Res. (2020) 30:215–21. 10.1007/s10286-020-00681-6 32246226 PMC7341538

[62] Udupa K Sathyaprabha TN Thirthalli J Kishore KR Lavekar GS Raju TR. Alteration of cardiac autonomic functions in patients with major depression: a study using heart rate variability measures. J Affect Disord. (2007) 100:137–41. 10.1016/j.jad.2006.10.007 17113650

[63] Rottenberg J. Cardiac vagal control in depression: a critical analysis. Biol Psychol. (2007) 74:200–11. 10.1016/j.biopsycho.2005.08.010 17045728

[64] Nahas Z Marangell LB Husain MM Rush AJ Sackeim HA Lisanby SH. Two-year outcome of vagus nerve stimulation (VNS) for treatment of major depressive episodes. J Clin Psychiatry. (2005) 66:1097–104. 10.4088/JCP.v66n0902 16187765

[65] Rottenberg J Clift A Bolden S Salomon K. RSA fluctuation in major depressive disorder. Psychophysiology. (2007) 44:450–8. 10.1111/j.1469-8986.2007.00509.x 17371497

[66] Greiner W Claes C Busschbach JJV von der Schulenburg J-MG. Validating the EQ-5D with time trade off for the German population. Eur J Health Econ. (2005) 6:124–30. 10.1007/s10198-004-0264-z 19787848

