# Supplementary material for: Autonomic Symptoms in Older Adults Are Common and Associated With Health-Related Quality of Life
Source: Front Neurol. 2021 Nov 23;12:757748. doi: 10.3389/fneur.2021.757748 (PMC8649956; doi:10.3389/fneur.2021.757748)
Supplement: Supplementary file 1 [file Data_Sheet_1.docx]

Supplementary Table 1. Frequency of parameters potentially associated with autonomic symptoms

|  | **yes** | **no** | **In the past** | **No data available** |
| --- | --- | --- | --- | --- |
| Physical activity | 481 (52%) | 435 (47%) |  | 12 (1%) |
| Obesity (BMI ≥30) | 188 (20%) | 694 (75%) |  | 46 (5%) |
| Tobacco use history | 73 (8%) | 429 (46%) | 416 (45%) | 10 (1%) |
| Hypertension | 409 (44%) | 508 (55%) |  | 11 (1%) |
| History of cardiovascular disease | 199 (21%) | 720 (78%) |  | 9 (1%) |
| Diabetes mellitus type 2 | 76 (8%) | 843 (91%) |  | 9 (1%) |
| Cataract | 194 (21%) | 724 (78%) |  | 10 (1%) |
| Glaucoma | 79 (8%) | 840 (91%) |  | 9 (1%) |
| Irritable bowel syndrome | 4 (0.4%) | 924 (99.6%) |  | 0 |
| Beta blockers | 182 (20%) | 784 (79%) |  | 12 (1%) |
| ACE inhibitors | 149 (16%) | 767 (83%) |  | 12 (1%) |
| Calcium channel blockers | 85 (9%) | 831 (90%) |  | 12 (1%) |
| Alpha-1 blockers | 5 (1%) | 911 (98%) |  | 12 (1%) |
| Antihistamines | 21 (2%) | 895 (97%) |  | 12 (1%) |

Physical activity was defined as 2 or more hours doing sport per week, no physical activity as less than 2 hours doing sport per week. BMI: Body Mass Index.

**Supplementary Table 2. Comparison of presence / proportion of potential confounders in those with low (< 75^th^ percentile, 0-16 points) and high (≥17 points) COMPASS 31 values**

|  | **COMPASS 31**  **<75^th^ percentile**  **n=696** | **COMPASS 31**  **>75^th^ percentile**  **n=232** | | **p** |
| --- | --- | --- | --- | --- |
| Physical activity | 380 (55%) | | 101 (45%) | 0.004* |
| Obesity (BMI ≥30) | 137 (21%) | | 51 (23%) | 0.435 |
| Tobacco use history | 54 (8%) | | 19 (8%) | 0.761 |
| Hypertension | 300 (44%) | | 109 (47%) | 0.444 |
| History of cardiovascular disease | 135 (20%) | | 64 (28%) | 0.010* |
| Diabetes mellitus type 2 | 57 (8%) | | 19 (8%) | 0.977 |
| Cataract | 130 (19%) | | 62 (28%) | 0.015* |
| Glaucoma | 49 (7%) | | 30 (13%) | 0.006* |
| Irritable bowel syndrome | 3 (0.4%) | | 1 (0.4%) | 1.000 |
| Beta blockers | 126 (18%) | | 56 (24%) | 0.094 |
| ACE inhibitors | 115 (17%) | | 34 (18%) | 0.460 |
| Calcium channel blockers | 62 (9%) | | 23 (10%) | 0.555 |
| Alpha-1 blockers | 1 (0.1%) | | 4 (1.7%) | 0.011* |
| Antihistamines | 13 (2%) | | 8 (4%) | 0.234 |
| EQ VAS | 80 | | 75 | <0.001* |

BMI: Body Mass Index; EQ VAS: Visual analogue scale of the EuroQol five-level version of EQ-5D.
